# Guide Wire Selection (Straight vs. Angled) in Endoscopic Retrograde Cholangiopancreatography Using a Normal Contrast Catheter Performed by a Trainee: A Single-Center Prospective Randomized Controlled Cross-Over Study

**DOI:** 10.3390/jcm12082917

**Published:** 2023-04-17

**Authors:** Takumi Maki, Atsushi Irisawa, Akira Yamamiya, Keiichi Tominaga, Yoko Abe, Koh Imbe, Koki Hoshi, Akane Yamabe, Ryo Igarashi, Yuki Nakajima, Kentaro Sato, Goro Shibukawa

**Affiliations:** 1 Department of Gastroenterology, Aizu Medical Center, Fukushima Medical University, 21-2 Kawahigashi Aizuwakamatsu, Fukushima 969-3482, Japan; m06094tm@jichi.ac.jp (T.M.);; 2 Department of Internal Medicine, Minamiaizu Prefectural Hospital, 14-1 Nagata Minamiaizu, Fukushima 967-0006, Japan; 3 Department of Gastroenterology, Dokkyo Medical University School of Medicine, 880 Kitakobayashi Mibu, Tochigi 321-0293, Japan

**Keywords:** endoscopic retrograde cholangiopancreatography, trainee, wire-guided cannulation

## Abstract

Introduction: Wire-guided cannulation (WGC) during endoscopic retrograde cholangiopancreatography (ERCP) is a selective biliary cannulation technique aimed at improving the successful selective biliary cannulation rate and reducing the rate of post-ERCP pancreatitis (PEP) incidence. This study aimed to evaluate the effectiveness of angled-tip guidewires (AGW) vs. straight-tip guidewires (SGW) for biliary cannulation via WGC by a trainee. Methods: We conducted a prospective, single-center, open-labeled, randomized, and controlled trial. Fifty-seven patients were enrolled in this study and assigned randomly to two groups (Group A to S and Group S to A). In this study, we started selective biliary cannulation via WGC with an AGW or an SGW for 7 min. If cannulation was unsuccessful, the other guidewire was used, and cannulation was continued for another 7 min (cross-over method). Results: The selective biliary cannulation success rate over 14 min was significantly higher with an AGW compared with an SGW over 14 min (57.8% vs. 34.3%, *p* = 0.04) and for the second 7-min segment (36.4% vs. 0%, *p* = 0.04). No significant difference was found for adverse events such as pancreatitis between the two guidewires. Conclusions: Our results suggest that an AGW is recommended for WGC performed by a trainee.

## 1. Introduction

Endoscopic retrograde cholangiopancreatography (ERCP) is a technically difficult endoscopic procedure with a high possible adverse event rate. Post-ERCP complications, especially post-ERCP pancreatitis (PEP), can sometimes prove fatal to patients. PEP is probably caused by premature zymogen activation in the acinar cells, pancreatic microvascular hypoperfusion, and ischemic injury [1]. Contrast imaging and rough guidewire manipulation are included. Prevention of PEP requires careful patient selection for ERCP, identification of individual risk factors, high-quality ERCP technique that includes guidewire manipulation, medications (e.g., non-steroidal anti-inflammatory drugs, nitrate drugs), and placement of a prophylactic pancreatic stent [2]. Among these, high-quality ERCP techniques have been pointed out as a particularly important factor related to PEP prevention. Performing selective bile duct cannulation rapidly is important to perform ERCP more safely. If all ERCP is performed by an expert, the incidence of PEP may decrease, but it is very important from an educational point of view to have trainees perform ERCP under intensive education while fully considering safety.

Efforts to achieve rapid bile duct cannulation include device development and cannulation techniques. They can be considered from various perspectives. Among the several techniques used for selective biliary cannulation during ERCP, wire-guided cannulation (WGC) during ERCP is a selective biliary cannulation technique aimed at improving rates of successful selective biliary cannulation and reducing rates of PEP incidence. Moreover, by reducing the direct load on the pancreatic duct, WGC can prevent PEP. Furthermore, by shortening the procedure time, it might be possible to avoid complications such as perforation, aspiration, and cholangitis [3,4]. Systematic reviews and meta-analyses have indicated that WGC is superior to conventional contrast methods for selective biliary cannulation [5,6]. However, it has been pointed out that a trainee performing ERCP tends to require a long time for the procedure, which might increase the rate of complication incidence [7]. Findings have demonstrated that the rates of complication caused by cannulation by a trainee using WGC are within the permissible range and that a high cannulation rate can be achieved within a certain timeframe [8]. According to those findings, WGC has been regarded as a useful procedure from various perspectives.

Often, WGC is performed using a straight-tip guidewire (SGW) loaded to a sphincterotome. However, no superiority or inferiority of using a conventional catheter and a sphincterotome has been demonstrated [9]. Regarding guidewire types, Vihervaara et al. reported finding no significant difference in the selective biliary cannulation success rate or the incidence rate of complications between an angled-tip guidewire (AGW) and an SGW [10]. Moreover, the time of selective biliary cannulation was reported as significantly shorter for an AGW than for an SGW [10]. However, that study investigated the performance of an expert operator. As mentioned above, it is an important issue to find out what kind of ingenuity is required for trainees to perform selective biliary cannulation safely and reliably. This research was planned to focus on the selection of GW in the WGC. We conducted a prospective, randomized controlled study to compare the effectiveness of AGW vs. SGW for biliary cannulation by a trainee.

## 2. Materials and Methods

### 2.1. Study Design

This study was conducted as a prospective, single-center, open-labeled, randomized, and controlled trial. Using sealed envelopes, we assigned patients to two groups before the procedure: Groups A to S (first, after using an AGW, if selective biliary cannulation was not achieved within 7 min, then an AGW was changed to an SGW. Cannulation was then continued for another 7 min.) and Groups S to A (first, after using an SGW, if selective biliary cannulation was not achieved within 7 min, then an SGW was changed to an AGW. Cannulation was then continued for another 7 min.) as the cross-over method. Double blinding was not possible since the tip-shape of the guidewire was naturally known during procedures. Difficult biliary cannulation was defined as unsuccessful cannulation over 5 min or 10 min [11,12]. If 10 min were set as the time limit, then we would have to perform biliary cannulation for a maximum of 20 min if doing a cross-over, which would increase the risk of complications for the patient. Therefore, we set the time limit at 7 min. The envelopes were assigned under strict management by a third person who was not involved in the procedure. The primary endpoint was the selective biliary cannulation success rate of each GW. The secondary endpoints were the biliary cannulation success rate of the first GW, the time of selective biliary cannulation by each GW, the pancreatic duct cannulation rate, the incidence rate of adverse events, and the final selective biliary cannulation success rate. Specific adverse events of interest were PEP according to the Cotton criteria [13], obvious GI bleeding, and perforation.

An earlier report presented the successful selective biliary cannulation rate for an SGW as 60% [14]. We hypothesized that an AGW would improve the successful selective biliary cannulation rate to 80%. Sample size calculations indicated a necessary total of approximately 134 patients to detect an increase in the selective biliary cannulation rate of WGC from 60% to 80% (a = 0.05, b = 0.8; two-tailed test).

### 2.2. Patients

Inclusion criteria in this study were: (1) patients undergoing endoscopic retrograde cholangiography or ERCP-related procedures (e.g., bile sampling, bile duct biopsy, lithectomy, or biliary drainage) at the Department of Gastroenterology, Aizu Medical Center, Fukushima Medical University; (2) patients aged 20 years or older; and (3) patients who provided written informed consent to participate in this study. On the other hand, the exclusion criteria were the following: (1) patients with a history of ERCP-related procedures (e.g., endoscopic sphincterotomies, stenting, stone removal); (2) patients with a history of intestinal reconstruction surgery; (3) patients scheduled for pancreatography; (4) patients with severe cardiopulmonary disease or shock, making endoscopy difficult; (5) patients with an interdiverticular papilla; and (6) pregnant or possibly pregnant women. The study protocol was approved by our institutional review board (registration number: 1802). The study was also registered with the University Medical Information Network (UMIN) (registration number: UMIN000013196). Written informed consent was obtained from all patients.

### 2.3. Endoscopic Procedure

All patients were administered pentazocine (15 mg) and midazolam (3–10 mg, depending on the patient status) for sedation and anesthesia before starting the endoscopic procedure. All patients received nafamostat mesylate (10 mg) by drip infusion for PEP prophylaxis during the endoscopic procedures. Procedures were conducted using side-viewing therapeutic duodenoscopy (JF-240, JF260V, and TJF260V; Olympus Medical Systems Corp., Tokyo, Japan). A standard double-lumen injection catheter (Tandem XL; Boston Scientific Japan, Tokyo, Japan) was used. A GW, either angled-tip or straight-tip (VisiGlide 2; Olympus Medical Systems Corp., Tokyo, Japan), was used. Because this study was designed to examine the selection of an appropriate GW in WGC, only a standard injection catheter was used without a sphincterotome that could be angled during cannulation.

The WGC was performed using the following method: (1) After looking straight at the duodenal papilla, we inserted a catheter that had been preloaded with a GW into the accessory channel of the duodenoscope. (2) We applied the catheter with a GW to the opening of the papilla, advanced the GW, and tried selective biliary cannulation by the GW alone. Selective biliary cannulation was performed carefully using a GW under fluoroscopy. When using an AGW, we did not rotate the AGW to search for the biliary tract opening. (3) When results suggested that a GW be cannulated into the biliary tract, we cannulated a catheter into the biliary tract. We then aspirated bile duct juice to confirm the achievement of selective biliary cannulation.

If selective biliary cannulation was not achieved within 7 min, then the GW was changed to the other type of GW. Cannulation was then continued for another 7 min. If selective biliary cannulation was not achieved within 14 min, then it was deemed unsuccessful. There were no restrictions on the method of selective biliary cannulation thereafter. Selective biliary cannulation with the assistance of contrast was deemed unsuccessful, even if it was within 14 min.

The ERCP operators (five trainees) had less than two years of experience in ERCP; either the operator or the assistant was capable of manipulating the GW. All the assistants were trainees, and they also have less than 2 years of experience in the manipulation of the GW. If selective biliary cannulation was not achieved within 14 min, then a trainer took over.

### 2.4. Statistical Analysis

The chi-square test, or Fisher’s exact test, was used to test for significant differences between categorical variables. A nonparametric Wilcoxon–Mann–Whitney test was used to compare differences in continuous and ordinal variables. Statistical analyses were performed using software (IBM SPSS Statistics ver. 27; IBM Corp., Armonk, NY, USA).

## 3. Results

### 3.1. Patient Characteristics

**Figure 1:**
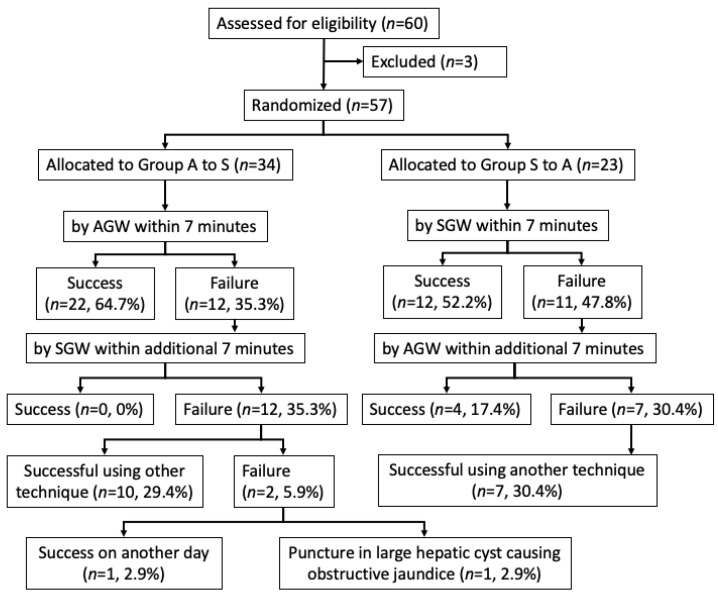
Consort chart of patient enrollment and randomization. AGW, angled-tip guidewire; SGW, straight-tip guidewire.

Baseline data for the two groups were well balanced (Table 1). Although we had the estimated number of 134 patients, enrollment in the study was discontinued after 60 patients were enrolled because the planned patient enrollment period had expired. One patient was excluded because we were unable to reach the duodenal papilla because of duodenal stenosis. Two patients were excluded because they did not undergo ERCP. Therefore, the 57 patients who required selective biliary cannulation of the native papilla were randomly assigned to two groups: 34 patients and 23 patients. Thirty-four patients were allocated to Groups A to S. Among this group, 22 patients had successful selective biliary cannulation. Twelve patients who had cannulation failure were switched to an SGW. All 12 patients had selective biliary cannulation failure. Then, the trainers took over the procedures. The remaining 10 of 12 patients had successful selective biliary cannulation after switching to other techniques: 6 patients used conventional contrast cannulation, 2 patients used the pre-cut method, 1 patient used the pancreatic duct guidewire placement method, and 1 patient used the randezvous method, which was performed via the transpapillary route combined with the percutaneous transhepatic route. The remaining patient had successful selective biliary cannulation on a different day. The other patient had a large hepatic cyst causing obstructive jaundice, for which percutaneous transhepatic drainage was performed. On the other hand, 23 patients were allocated to Groups S to A. Among this group, 12 patients had successful selective biliary cannulation. Eleven patients who had cannulation failure were switched to an AGW. This treatment was successful in four patients. Then, the trainers took over the procedures. Regarding the remaining 7 patients, all had successful selective biliary cannulation after switching to other techniques (Figure 1); 3 patients used the conventional contrast cannulation, 2 patients used the pre-cut method, and 2 patients used the pancreatic duct guidewire placement method. The numbers of cases treated by each trainee in this study were almost equal.

### 3.2. Successful Biliary Cannulation Rate

All the assistants who manipulated a GW were trainees with less than 2 years of experience. The selective biliary cannulation success rate using an AGW for the 14 min was 57.8% (26/45) whereas the selective biliary cannulation success rate was 34.3% (12/35) using an SGW. The selective biliary cannulation success rate was significantly higher with an AGW than with an SGW (*p* = 0.04) (Table 2). The selective biliary cannulation success rate using a single GW for the first 7 min was 64.7% (22/34) in Group A to S and 52.1% (12/23) in Group S to A (*p* = 0.34) (Table 3). Of the 12 patients who had cannulation failure for the first 7 min in Group A to S, all patients had unsuccessful selective biliary cannulation after switching to an SGW (0%). In Group S to A, 11 patients who had cannulation failure for the first 7 min were switched to an AGW; 4 patients had successful selective biliary cannulation (36.4%). The successful selective biliary cannulation rate for the second 7-minute segment was significantly higher with an AGW than with an SGW (*p* = 0.04). Of the 12 patients with cannulation failures for 14 min in Groups A to S, 10 had successful selective biliary cannulation after switching to other techniques. The final successful selective biliary cannulation rate was 94.1% (32/34). In Groups S to A, 7 patients who had cannulation failure for the 14 min had successful selective biliary cannulation after switching to other techniques. The final successful selective biliary cannulation rate was 100% (23/23) (Figure 1).

### 3.3. Successful Selective Biliary Cannulation Time

The median successful selective biliary cannulation time that elapsed when using an AGW for the 14 min was 120 s (10–420 s, 26 cases). The time spent using an SGW for the 14 min was 100 s (60–390 s, 12 cases). No significant difference was found between the GWs (*p* = 0.49) (Table 2). The median cannulation time using a single GW for the first 7 min was 101 s (10–420 s, 22 cases) for Groups A to S and 100 s (60–390 s, 12 cases) for Groups S to A (*p* = 0.87) (Table 3).

### 3.4. Incidence Rate of Post-ERCP Pancreatitis

The pancreatic duct cannulation rate using a single GW within 14 min was 22.7% (5/22) in group A to S; it was 25.0% (3/12) in Groups S to A. The incidence rate of PEP in Groups A to S, in which selective biliary cannulation was achieved by an AGW without cross-over, was 4.5% (1/22). The rate for group S to A, in which selective biliary cannulation was achieved using an SGW without cross-over, was 8.3% (1/12). No significant difference was found between these results (*p* = 0.59) (Table 4). All PEP cases were mild based on the Cotton classification. Including cases that exceeded 14 min, the PEP incidence rate was 7.0% (4/57) in all cases, 8.8% (3/34) in Groups A to S, and 4.3% (1/23) in Groups S to A. No significant difference was found among the incidence rates of PEP when using only an AGW, only an SGW, or both GWs (Table 5).

In Groups A to S, PEP occurred in three cases. One of the three cases was cannulated in the biliary tract by the pancreatic duct guide wire method after cross-over from an AGW to an SGW. Another one was cannulated in the biliary tract by EPST after cross-over from an AGW to an SGW. The last case was cannulated in the biliary tract with an AGW. In all cases, pancreatography or pancreatic duct cannulation was performed. In Group S to A, one case was cannulated in the biliary tract with an SGW.

## 4. Discussion

Even with the improved accuracy of MRCP, ERCP still plays an important role in the diagnosis and treatment of pancreatobiliary disease. To improve the successful cannulation rate and prevent post-ERCP complications, using several optimal techniques and selecting optimal devices is important. Even among them, the selection of GW is important in ERCP. Generally speaking, an SGW might be used if the purpose of an ERCP is to remove choledocholithiasis. However, an AGW is useful when inserting a GW for perihilar cholangiocarcinoma that requires a search for a stenosed duct. The original method of WGC was to load an SGW onto a sphincterotome and perform selective biliary cannulation. If a search is necessary after cannulation, then an SGW must be changed to an AGW, which is both troublesome and costly. Two reports [9,15] have described that no significant difference was found between the successful selective biliary cannulation rate and the incidence rate of PEP between the standard cannulation technique using contrast medium injection and WGC in randomized trials, systematic reviews, or meta-analyses [3,4,6,9,16,17,18], and that WGC has a significantly higher successful selective biliary cannulation rate and a significantly lower incidence rate of PEP. On the other hand, although ERCP performed by trainees is recognized as a risk factor for PEP, the trainees must also be provided with solid on-the-job training. Based on those findings and our own, WGC is presumed to be suitable for a procedure performed by a trainee. Against this background, this trial was conducted. Kurita et al. [19] reported the selective biliary cannulation rate of trainees performing WGCs. They conducted a single-center prospective randomized controlled study and compared a rotatable sphincterotome with a conventional sphincterotome to assess their associated selective biliary cannulation rates for WGC performed by trainees. Selective biliary cannulation success rates were 68% (68/100) for a rotatable sphincterotome and 62% (62/100) for a conventional sphincterotome. The two groups did not differ significantly. However, Kurita et al. did not compare an AGW and an SGW for WGC. The present report is the first of a study comparing an AGW and an SGW in WGC by a trainee. We were able to evaluate what procedure WGC is suitable for the trainee.

From our trial, no difference was found in successful selective biliary cannulation rates between an AGW and an SGW during 0–7 min. However, the successful selective biliary cannulation rate of an AGW was higher than that of an SGW within 14 min and during 7–14 min. This finding suggests that a case that has had successful selective biliary cannulation by WGC using an SGW can have selective biliary cannulation by WGC using an AGW. In other words, using an AGW by WGC as the first GW might lead to a higher rate of successful selective biliary cannulation than when using an SGW. The original WGC is to load an SGW to a sphincterotome, adjust the direction with a catheter, and cannulate into the biliary tract, but in ERCP performed by a trainee, who might have insufficient catheter operation experience, the tip angle of an AGW might contribute to selective biliary cannulation. Furthermore, we used a standard injection catheter that could not be angled with a knife. An AGW could be adjusted to the angle of the biliary tract compared with an SGW in cases where a standard injection catheter is used. Therefore, the combination of an AGW and a standard injection catheter was considered capable of performing cannulation similarly to that of an SGW and a sphincterotome with adjustable angulation. Vihervaara et al. [10] reported the selective biliary cannulation rates by WGC obtained with an AGW or an SGW. They conducted a single-center prospective randomized controlled study. Patients were allocated to two groups: an AGW group and an SGW group. In the AGW group, an AGW was used. An SGW was used in the SGW group. A 5-Fr cannula was used in their study, not a sphincterotome. Their study protocol did not include crossover to the other GW arm if the randomized GW cannulation proved unsuccessful within the protocol time. In these cases, the endoscopists proceeded with the cannulation according to their personal preferences and evaluations. Selective biliary cannulation success rates were 60% (42/70) for an AGW and 65% (54/83) for an SGW by four experienced endoscopists and one trainee with experience of approximately 50 ERCPs. These selective biliary cannulation success rates were similar to our results. Findings from this trial suggest that the use of trainers or expert operators led to a difference in the results. Specifically, for an expert, the type of GW used for WGC is not expected to affect the rate of successful selective biliary cannulation. Our examination of whether the subsequent procedure can be completed with a GW used for selective biliary cannulation revealed that, in two cases of selective biliary cannulation using an SGW, seeking of the intrahepatic bile duct after cannulation was necessary, followed by replacement with an AGW. For reducing WGC costs by reducing the number of devices to be used and by reducing the procedures after selective biliary cannulation, using an AGW first might be useful, irrespective of the operator’s ability.

Some disagreement persists about the benefits of WGC. Numerous reports have described a low incidence rate of PEP. In our trial, the overall incidence rate of PEP in patients who completed the cross-over study within the cross-over study time (within 14 min) was 5.4% (4.5% in groups A to S; 6.3% in groups S to A), which is almost identical to findings reported earlier. Furthermore, no significant difference was found between both types of GW. Although our number of procedures was small, this is an important finding that permits the training of trainees without increasing the risk of harm to our patients. When using an AGW, the axial rotation might be applied to seek the biliary tract opening, but this action imposes a load on the papillary opening and might cause papilledema and PEP. In our trial, the PEP incidence rate might not have been high because such rotation was not performed. When performing WGC using an AGW, this point must be addressed.

For the successful selective biliary cannulation rate for WGCs conducted by a trainee, Theodor et al. [14] reported the final selective biliary cannulation success rate by trainees performing WGCs for the first time as 61.8% (314/822). This result was not significantly different from the selective biliary cannulation success rate found in our trial. The PEP incidence rate was 3% (25/822), which was also similar to our results. To date, no report has described a comparison of WGCs performed by trainees using GWs of different shapes. Our trial results are expected to help trainees choose a strategy for selective biliary cannulation in ERCP.

Our trial had several limitations, the first of which was the small sample size. Although we had the estimated number of 134 patients, enrollment in the study was discontinued at the time 60 patients were enrolled because the planned patient enrollment period had expired. As a result, the patient allocation by randomized control was not completed. The study period expired before enrolling 134 patients; therefore, there was a gap in the number of patients enrolled in the two groups despite the fact that we randomized the 60 patients who enrolled in this study. Nevertheless, the findings do indicate similar selective biliary canulation success rates and PEP incidence rates for trainees, whether using an AGW or an SGW. Moreover, these selective biliary cannulation success rates and PEP incidence rates were not so different from those described in earlier reports. These results might be regarded as receiving a certain appreciation. Secondly, randomization for our trial was performed using the envelope method. If the envelope method is misused, then concealment of allocation is not guaranteed, and selection bias might therefore occur. Nevertheless, we strictly managed and allocated envelopes using a third person who was uninvolved in the procedure. Therefore, this shortcoming of our trial does not lead to selection bias [20]. Additionally, the definition of difficult biliary cannulation set at 7 min might be somewhat ambiguous. Because this trial was begun in 2014, we referred to the European Society for Gastroenterological Endoscopy (ESGE) guidelines of 2014 and set the time limit to 7 min. The ESGE guideline for 2014 has since been updated in 2020. The definitions of difficult biliary intubation are the same in the ESGE guidelines of 2014 and 2020 [21].

## 5. Conclusions

We infer from our trial findings that an AGW is recommended for WGC performed by a trainee with a trainee assistant, even for those who might have insufficient catheter and GW operation experience. Results suggest that the selection of an AGW is useful to meet needs after selective biliary cannulation and to manage GW costs. However, it is difficult to say that sufficient evidence was obtained, partly because the number of subjects in this study was small. Considering that a large-scale study is necessary.

## Figures and Tables

**Table 1:** Patient characteristics.

Characteristics	Group A to S	Group S to A	*p* Value
Number of patients	34	23	
Median age (range) (years)	76 (34–99)	79 (53–94)	0.52
Sex: male/female	15/19	9/14	0.71
Periampullary diverticulum	3	3	0.67
Choledocholithiasis	14	9	0.98
Cholangiocarcinoma	9	1	0.7
Gallbladder cancer	0	5	0.07
Pancreatic cancer	1	0	1
Cholangitis	3	1	1
Cholecystitis	2	2	0.64
Others (benign bile duct stricture) *	3	4	0.42
Others (malignant bile duct stricture) †	2	1	1

* Benign bile duct stricture includes hepatic cyst, chronic pancreatitis, pancreatic tumor, cholangio tumor, and gallbladder debris. † Malignant bile duct stricture includes hepatocellular carcinoma recurrence in hilar.

**Table 2:** Successful selective biliary cannulation rate and time for cannulation for 14 min.

Variable	AGW	SGW	*p* Value
Number of patients	45	35	
Success rates of each GW (%)	26 (57.8)	12 (34.3)	0.04
Time for cannulation, s (range)	120 (10–420)	100 (60–390)	0.49

GW—guidewire; AGW—angled-tip guidewire; SGW—straight-tip guidewire.

**Table 3:** Successful selective biliary cannulation rate and time for cannulation for the first 7 min.

Variable	AGW	SGW	*p* Value
Number of patients	34	23	
Success rates of each GW (%)	22 (64.7)	12 (52.1)	0.34
Time for cannulation, s (range)	101 (10–420)	100 (60–390)	0.87

GW—guidewire; AGW—angled-tip guidewire; SGW—straight-tip guidewire.

**Table 4:** Incidence rate of PEP at 14 min.

Variable	Only AGW	Only SGW	*p* Value
PEP rate (%)	1/22 (4.5%)	1/12 (8.3%)	0.59

AGW—angled-tip guidewire; SGW—straight-tip guidewire; PEP—post ERCP pancreatitis.

**Table 5:** Final PEP rates.

Variable	PEP	*p* Value			
only AGW	1/22 (4.5%)	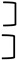	0.59	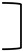	
only SGW	1/12 (8.3%)				0.52
AGW and SGW	2/23 (8.7%)		0.73		

AGW—angled-tip guidewire; SGW—straight-tip guidewire; PE—post ERCP pancreatitis.

## Data Availability

Data are accessible upon request from the corresponding authors.
